# Mechanisms of nasal high flow therapy in newborns

**DOI:** 10.1152/japplphysiol.00871.2019

**Published:** 2020-02-20

**Authors:** Pavel Mazmanyan, Mari Darakchyan, Maximilian I. Pinkham, Stanislav Tatkov

**Affiliations:** ^1^Department of Neonatology, Yerevan State Medical University, Yerevan, Armenia; ^2^Fisher & Paykel Healthcare, Auckland, New Zealand

**Keywords:** CPAP, nasal high flow, neonate, ventilation, work of breathing

## Abstract

In newborns, it is unclear how nasal high flow (NHF) generates positive airway pressure. In addition, the reported benefits of NHF such as reduction in work of breathing may be independent of airway pressure. The authors hypothesized that during NHF the area of leak and the flow determine airway pressure and that NHF can reduce the required minute ventilation to maintain gas exchange. In response to NHF, pressure was measured in the upper airways of 9 newborns and ventilation was measured in another group of 17 newborns. In a bench model, airway pressures were measured during NHF with different prong sizes, nare sizes, and flows. The airway pressures during 8 L/min NHF were greater when a larger cannula versus a smaller cannula was used (*P* < 0.05). NHF reduced minute ventilation in 16 of 17 neonates, with a mean decrease of 24% from a baseline of 0.66 L/min (SD 0.21) (*P* < 0.001), and was unrelated to changes in airway pressure; arterial oxygen saturation by pulse oximetry ($\text{S}{\text{p}}_{{\text{O}}_{2}}$) and tissue CO_2_ were unchanged. In the bench model, the airway pressure remained <2 cmH_2_O when <50% of the “nare” was occluded by the prongs. As the leak area decreased, because of a smaller nare or a larger cannula, the airway pressure increased exponentially and was dependent on flow. In newborns NHF using room air substantially reduced minute ventilation without affecting gas exchange irrespective of a decrease or an increase of respiratory rate. NHF generates low positive airway pressure that exponentially increases with flow and occlusion of the nares.

**NEW & NOTEWORTHY** In healthy newborns, nasal high flow (NHF) with room air reduced minute ventilation by one-fourth without affecting gas exchange but, in contrast to adults, produced variable response in respiratory rate during sleep. During NHF, pressure in the upper airways did not exceed 2 cmH_2_O at 8 L/min (3.4 L·min^−1^·kg^−1^) and was unaffected by opening of the mouth. NHF can generate higher pressure with larger prongs that decrease the leak around the cannula or by increasing the flow rate.

## INTRODUCTION

Nasal high flow (NHF) provides respiratory support for spontaneously breathing patients across all ages. It is used in newborns because of its ease of use and ability to accommodate feeding and contact with parents (4, 8). However, some studies have found continuous positive airway pressure (CPAP) to be superior to NHF in neonates of low birth weight (17, 24, 29). A better understanding of the mechanisms can help in the decision making for NHF application as both a primary respiratory support and complementary therapy.

Respiratory distress syndrome in preterm infants is characterized by atelectasis, and elevated positive end-expiratory pressure (PEEP) is known to reduce this (28, 34). In neonates with respiratory problems, insufficient PEEP may not improve atelectasis and gas exchange will be impaired, but if the pressure is too high then cardiac output may be compromised. CPAP delivers a constant pressure at a setting determined by the operator, which is often 5–6 cmH_2_O in neonates. In contrast, NHF is a flow-controlled therapy, and the positive airway pressures that are generated remain unclear. During NHF, the nasal prongs do not occlude the nares; therefore, determining the airway pressures is difficult. The available data are conflicting (12, 15, 16, 30, 32, 33, 36), and it is important to define how airway pressures are generated during NHF.

The fresh gas flow into the nasopharynx from NHF leads to dilution and purging of expired gas from the nasal cavity (21, 22); this causes a reduction in the rebreathing of gas rich in CO_2_ and low in O_2_ (27). The reduction in the rebreathing of CO_2_ is considered to be the primary mechanism by which NHF reduces minute ventilation and work of breathing in adults (3, 27). However, the ventilation responses to NHF have not been investigated in neonates. The control of breathing may be different in neonates compared with adults, and the breathing responses to NHF need to be understood to improve the application of the therapy.

The authors hypothesized that during NHF the area of leak and the flow determine airway pressure and that NHF can reduce the required minute ventilation to maintain gas exchange.

## METHODS

The clinical part of the study was conducted on neonates born in the Research Center of Maternal and Child Health Protection maternity department in Yerevan, Armenia. After approval by the Ethics Committee, infants from the postnatal and special care units were recruited after oral and written parental consent.

### Study Population

Healthy newborns were eligible for inclusion if they were full or near term and 0–3 days old. Newborns were excluded if they were unhealthy or had a known respiratory, cardiac, or gastrointestinal anomaly. The baseline characteristics are shown in Table 1.

**Table 1.** Anthropometric data

	Pressure Study	Ventilation Study
*n*	9	20
Gestation, wk	37.1 ± 1.4	39.1 ± 1.5
Postnatal age, day	1.1 ± 0.4	1.3 ± 0.5
Birth weight, g	2,573 ± 477	3,090 ± 441
Length, cm	48.4 ± 2.2	50.5 ± 2.1
Male, *n* (%)	5 (56%)	12 (60%)

Data are means ± SD.

### Protocols

#### Clinical section.

##### nasopharyngeal pressures in neonates during nhf.

Two microtip (diameter 760 µm) catheter transducer probes (Mikro-Cath; Millar, United States) were inserted up to 15 mm into each nare; the simultaneous pressure measurements were averaged to provide the pressure within the nasal cavity. Ventilation was monitored with calibrated respiratory inductance plethysmography (RIP) (Respitrace QDC; Viasys, United States) as described previously (23). Briefly, calibration of RIP was performed with a precision spirometer (FE141) and respiratory flow head (MLT10L) (ADInstruments, New Zealand) through a mask used for bubble CPAP (bCPAP) at the beginning and end of each experiment. The spirometer was calibrated with a 3-L syringe (Hans Rudolph, Inc., United States). Continuous video monitoring was used to confirm the open/closed position of the mouth.

A pilot study in six newborns, gestational age (GA) 36 wk (SD 2) and body weight 2,516 g (SD 443), was performed to determine the pressures and safety of different flow rates (19). NHF rates of 5 L/min and 10 L/min were applied via the larger cannula (OPT316; Fisher & Paykel Healthcare Ltd., New Zealand). The study indicated that a flow rate of up to 4 L·min^−1^·kg^−1^ in near-term neonates was tolerated and safe in regard to the maximum generated pressure to perform a longer physiological study to investigate ventilation changes.

The nine newborns included in the analysis had a GA of 37.1 wk (SD 1.4) and a birth weight of 2,573 g (SD 477). NHF was applied at 8 L/min of air (AIRVO2) (Fisher & Paykel Healthcare Ltd., New Zealand) through smaller [outer diameter (OD) 3.17 mm and inner diameter (I.D) 1.55 mm] and larger (OD 3.82 mm and ID 2.35 mm) nasal cannulas (OPT314 and OPT316; Fisher & Paykel Healthcare Ltd., New Zealand). CPAP was used as a control, with pressure set to 5 cmH_2_O and biased flow of 8 L/min of humidified air. It was delivered via a bCPAP system through a nasal mask. Baseline values were obtained while no therapy was applied. The response to each intervention was recorded with the mouth closed and open; if necessary, the mouth was held in the required position. Interventions were applied in randomized order for 1–2 min each.

##### ventilation in neonates during nhf.

A total of 20 newborns were enrolled with a GA of 39.1 wk (SD 1.5) and a birth weight of 3,090 g (SD 441). The ventilation response to NHF was measured with the calibrated RIP. Transcutaneous carbon dioxide (tcCO_2_) and tissue oxygen (tcO_2_) tensions (Tosca TCM4; Radiometer, Denmark), arterial oxygen saturation by pulse oximetry ($\text{S}{\text{p}}_{{\text{O}}_{2}}$), and heart rate (Masimo, United States) were recorded continuously. EEG (Alice Polysomnography System; Philips, United States) was used to determine the sleep/awake status. NHF was applied at 8 L/min of air with AIRVO2 via the larger nasal cannula (OPT316; Fisher & Paykel Healthcare Ltd., New Zealand). NHF with room air only and no supplemental oxygen was applied for 30 min to each newborn. Baseline measurements were obtained while the baby was not wearing an interface and was not receiving therapy.

#### Bench section: bench model of pressure generation by NHF.

To understand how NHF generates positive airway pressures, a bench model was used. A chamber with two adjacent orifices to fit NHF cannula served as a model of nasal valve area in the upper airway; hole diameters were 5.5, 5.0, 4.5, and 4.0 mm. The “nare” sizes were chosen so that the airway pressures generated by NHF were similar to the measured pressures in neonates in the clinical section of this study. An artificial lung simulator (ASL5000; Ingmar Medical, United States) generated breathing flows through a separate part of the chamber and was calibrated to represent the breathing of a term neonate (resistance, 40 cmH_2_O·L^−1^·s; compliance, 4 mL/cmH_2_O; breaths per minute, 50). During simulated breathing, peak inspiratory pressure (PIP), peak expiratory pressure (PEP), and the dynamic range (PEP − PIP) were measured. Measurements were acquired during the different permutations of NHF rate, nare diameter, and cannula size and static condition or simulated breathing.

### Data Analysis

All data were recorded with an ADI PowerLab (ADInstruments, New Zealand) and then analyzed with LabChart software (ADInstruments, New Zealand).

Prism v.8 (GraphPad Software, San Diego, CA) was used to perform the statistical analysis. In the clinical part of the pressure study, one-way ANOVA with Dunnett’s test was performed to compare intervention with baseline. To compare interventions, a two-way ANOVA with Tukey’s post hoc test was performed. In the ventilation part of the study, the comparison between baseline (no NHF) and intervention (NHF) variables was made with a paired Student’s *t* test. In the bench-top study, to predict PEEP and the dynamic range of pressure during the application of NHF, an exponential growth curve was fitted to the data. Results are expressed as means (SD) unless otherwise stated.

## RESULTS

### Clinical

#### Nasopharyngeal pressures in neonates during NHF.

**Fig. 1.**
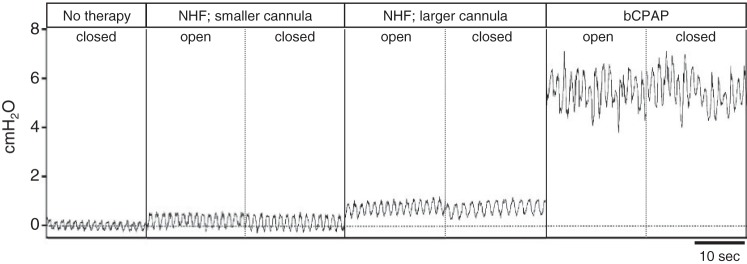
Raw recordings of the pressure (cmH_2_O) in the nasal cavity of a spontaneously breathing neonate during no therapy, nasal high flow (NHF) at 8 L/min via smaller and larger cannulas, and bubble continuous positive airway pressure (bCPAP) set to 5 cmH_2_O. Recordings were obtained with mouth closed and mouth open during each therapy.

**Fig. 2.**
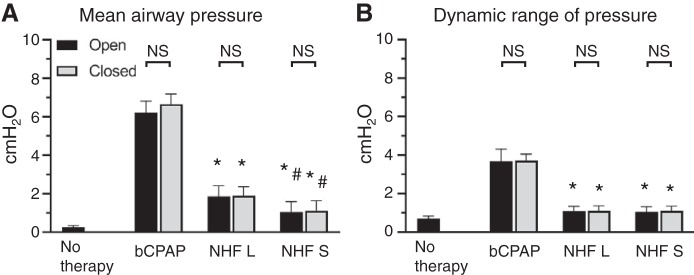
Bar graphs showing the mean (SD) airway pressure (*A*) and dynamic range of pressure (peak expiratory pressure − peak inspiratory pressure) (*B*) during no therapy, bubble continuous positive airway pressure (bCPAP) set to 5 cmH_2_O, and nasal high flow (NHF) set to 8 L/min via a larger cannula (NHF L) and a smaller cannula (NHF S) with mouth open and mouth closed. *Significant difference vs. bCPAP; *P* < 0.05; ^#^significant difference vs. NHF L, *P* < 0.05. NS, not significant.

Figure 1 shows example recordings of nasal pressures. NHF and bCPAP generate positive airway pressure and increase the pressure swings during the breathing cycle. As shown in Fig. 2, the open/closed position of the mouth did not affect the pressure values. bCPAP set to 5 cmH_2_O with a bias flow rate of 8 L/min generated a mean pressure of 6.23 cmH_2_O (SD 0.59) with the mouth open and 6.67 cmH_2_O (SD 0.51) with mouth closed. bCPAP increased the dynamic range of pressures, PEP – PIP, to 3.67 cmH_2_O (SD 0.65) (mouth open) and 3.72 cmH_2_O (SD 0.34) (mouth closed) from 0.75 cmH_2_O (SD 0.21) during no therapy (*P* < 0.05). NHF at 8 L/min increased the mean pressure to 1.86 cmH_2_O (SD 0.55) with mouth open and 1.89 cmH_2_O (SD 0.47) with mouth closed via the large cannula and 1.05 cmH_2_O (SD 0.54) with mouth open and 1.12 cmH_2_O (SD 0.51) with mouth closed via the small cannula (*P* < 0.05). During NHF, the dynamic range of pressure was greater than baseline and less than bCPAP. The ventilation responses during this experiment were highly variable, and no statistically significant differences were observed (Table 2). There was no correlation between the change in ventilation parameters and the change in airway pressures.

**Table 2.** Ventilation parameters in 9 neonates during bCPAP set to 5 cmH_2_O and NHF at 8 L/min delivered via a larger and a smaller cannula

		bCPAP		NHF—Larger Cannula		NHF—Smaller Cannula	
	Baseline	Mouth closed	Mouth open	Mouth closed	Mouth open	Mouth closed	Mouth open
Minute ventilation, mL/min	738 ± 256	650 ± 170	600 ± 203	491 ± 211	520 ± 172	584 ± 186	561 ± 262
Respiratory rate, breaths/min	52 ± 9	55 ± 15	47 ± 14	42 ± 17	41 ± 12	53 ± 16	35 ± 16

Data are means ± SD. Recordings were obtained with mouth open and mouth closed. bCPAP, bubble continuous positive airway pressure; NHF, nasal high flow.

#### Ventilation in neonates during NHF.

**Fig. 3.**
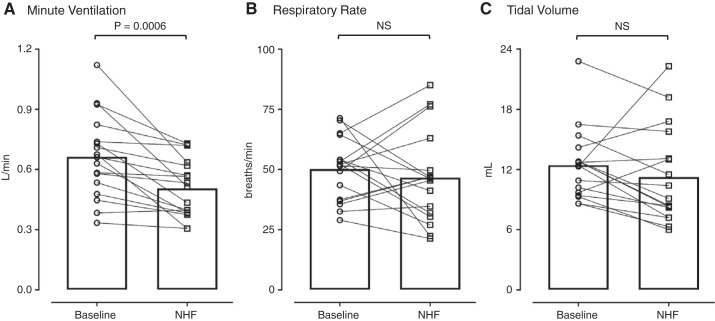
Ventilation parameters during the application of nasal high flow (NHF) at 8 L/min in sleeping neonates. NHF reduced minute ventilation (*A*) in all neonates, but the respiratory rate (*B*) and tidal volume (*C*) responses were variable. The group means are shown as bar graphs, and individual data points are shown by lines. NS, not significant.

Of the 20 neonates, only 3 remained awake long enough for data analysis during wakefulness; therefore, only ventilation during sleep was analyzed. NHF of 8 L/min reduced minute ventilation in 16 of 17 newborns, with a mean decrease of 158 mL/min from a baseline of 664 mL/min (SD 207) (*P* = 0.0006) (Fig. 3). The 24% reduction in minute ventilation was associated with variable responses in respiratory rate and tidal volume without affecting gas exchange or heart rate (Table 3).

**Table 3.** Ventilation parameters in 17 neonates before and during application of NHF at 8 L/min

	No NHF	NHF 8 L/min
Minute ventilation, mL/min	664 ± 207	506 ± 145*
Respiratory rate, breaths/min	50 ± 3	47 ± 5
$\text{S}{\text{p}}_{{\text{O}}_{2}}$, %	98 ± 1.9	98 ± 2.3
Heart rate, beats/min	128 ± 9	128 ± 8
Tissue O_2_, mmHg	45 ± 12	51 ± 11
Tissue CO_2_, mmHg	36 ± 5	37 ± 5

Data are means ± SD. NHF, nasal high flow; $\text{S}{\text{p}}_{{\text{O}}_{2}}$, arterial oxygen saturation by pulse oximetry.

* *P* = 0.0006.

**Fig. 4.**
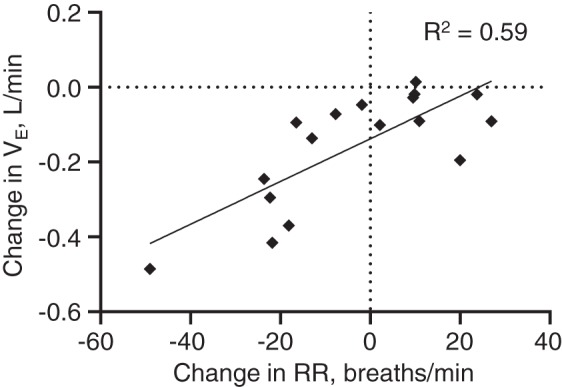
Relationship between change in respiratory rate (RR, breaths/min) and change in minute ventilation (V̇e, L/min) in response to nasal high flow of 8 L/min in 17 neonates. A greater decrease in respiratory rate was associated with a greater reduction in V̇e.

In response to NHF, a greater decrease in respiratory rate was associated with a greater reduction in the minute ventilation (*R*^2^ = 0.59; Fig. 4). No significant effects of NHF on inspiratory time (Ti), expiratory time (Te), or Ti/Te were observed.

### Bench: Pressure Generation by NHF in a Bench Model

**Fig. 5.**
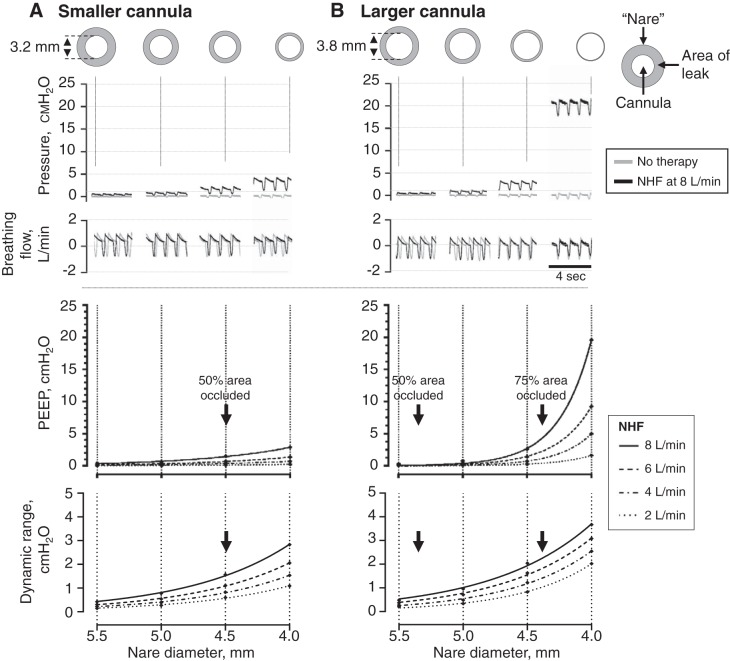
Results from the bench experiment. *Top*: raw recordings of the chamber pressure (cmH_2_O) and breathing flow (L/min) in the bench model; the gray line indicates recordings during no nasal high flow (NHF), and the black line indicates recordings during NHF at 8 L/min via the smaller cannula (*A*) or the larger cannula (*B*). *Bottom*: positive end-expiratory pressure (PEEP, cmH_2_O) and dynamic range of pressure (maximum − minimum, cmH_2_O) at different flow rates and nare diameters. The symbols indicate the raw data, and the lines indicate the exponential growth curve created from the data. The figure demonstrates that the pressure and pressure swings increase as the area of “nare” occlusion by the NHF cannula increases.

Figure 5 demonstrates the pressures in the model during no therapy or 8 L/min NHF. For the same nare size, the pressure is higher when NHF is delivered via the larger cannula. During NHF, pressure remained below 2 cmH_2_O until the combination of the larger cannula (OD 3.82 mm) and the orifice diameter of 4.0 mm resulted in an occlusion of 91% of the area, when pressure was ~20 cmH_2_O. During a breathing cycle, the swings in pressure between the peak of inspiration and peak of expiration increase as the leak around the prongs reduces, as either the orifice diameter reduces or the cannula size increases.

The PEEP remained < 2 cmH_2_O when <50% of the “nare” area was occluded by the prongs. As the total occlusion increased, the PEEP increased exponentially. There was a nonlinear effect of flow on pressure, an increase in the flow rate resulted in an exponential increase in pressure, and the effect size was dependent on the area of leak. The occlusion of 50% of total area of a smaller nare (Fig. 5*A*) generated a greater PEEP compared with the occlusion of 50% of total area of a larger nare (Fig. 5*B*) because of differences in the area of leak around the prongs; a smaller leak resulted in greater PEEP. The dynamic range of the pressures, maximum − minimum, increased exponentially as the area of the leak was reduced.

## DISCUSSION

NHF at 8 L/min in near- or full-term neonates generated pressures of <2 cmH_2_O in the nasal cavity. An increase in the size of the prongs, and therefore the area of the nasal occlusion, can increase the pressure. In the second set of experiments, NHF at 8 L/min reduced the minute ventilation by ~24% while gas exchange remained constant.

In the present study, the ventilation responses to 30 min of NHF in sleeping newborns were measured with calibrated RIP. Neonates spend the majority of their time sleeping, and that is therefore the most common setting for NHF application. In response to NHF, minute ventilation was reduced in 16 of 17 sleeping newborns, with a group mean decrease of 24% and no change in gas exchange. The present findings are consistent with reduced rebreathing from dead space (27). The 24% decrease in minute ventilation is similar to the response seen in sleeping adults of a reduction of ~20% (3, 23, 27). The extrathoracic dead space is proportionally larger in newborns (26), and therefore the dead space clearance by NHF may result in a greater decrease in the total ventilation required for gas exchange compared with adults (27). This is the first study to show that minute ventilation is consistently reduced in neonates in response to NHF, which may be a mechanism by which NHF can reduce the work of breathing.

Neonates have an immature control of breathing that results in irregular breathing patterns (6). Physiological measurements in neonates are difficult, and little is understood regarding the breathing responses to NHF. In the clinical setting the respiratory rate can be measured, whereas the tidal volume is not. In the present study, a greater decrease in the respiratory rate was associated with a greater reduction in minute ventilation (*R*^2^ = 0.59). However, although minute ventilation decreased in almost all neonates, an increase in the respiratory rate was observed in 8 of 17 neonates (47%). The results indicate that the change in the respiratory rate during NHF in neonates should be considered with caution; an increase in the respiratory rate may still be associated with a reduction in the required minute ventilation. Sleeping adults maintain a constant respiratory rate but decrease the tidal volume in response to NHF (2); the decrease in tidal volume is in response to a reduction in the rebreathing of expired CO_2_ (27). The present study shows different mechanisms of the decrease in minute ventilation in sleeping neonates. The study was performed in healthy subjects, and it is possible that the breathing responses in respiratory failure may be different. Previous findings show that preterm neonates have a sensitivity to inspired CO_2_ similar to that in term newborns (1, 5, 9); therefore, it is expected that NHF would result in a decrease of minute ventilation in neonates of varying GAs, but future studies are required.

The findings demonstrate that the pressure that is generated by NHF in neonates is unlikely to exceed 2 cmH_2_O. NHF was delivered at 8 L/min in all neonates regardless of size and weight. The mean flow rate of 3.43 L·min^−1^·kg^−1^ (SD 0.63) in the first part of the study, when airway pressure was measured, is higher than the 2 L·min^−1^·kg^−1^ that is typically prescribed (35). The relatively high flows resulted in airway pressures < 2 cmH_2_O and suggest that higher flows can be safe in neonates. However, low positive airway pressure generated during the application of NHF may, in part, explain unfavorable clinical outcomes shown in some studies when comparing to CPAP in preterm neonates with respiratory distress (24, 29). Previous research has demonstrated that the airway pressure during NHF is flow dependent: higher flow generates greater pressure (31). Future research should explore the effect of flow rate and the pharyngeal pressures during NHF on clinical outcomes in preterm neonates.

**Fig. 6.**
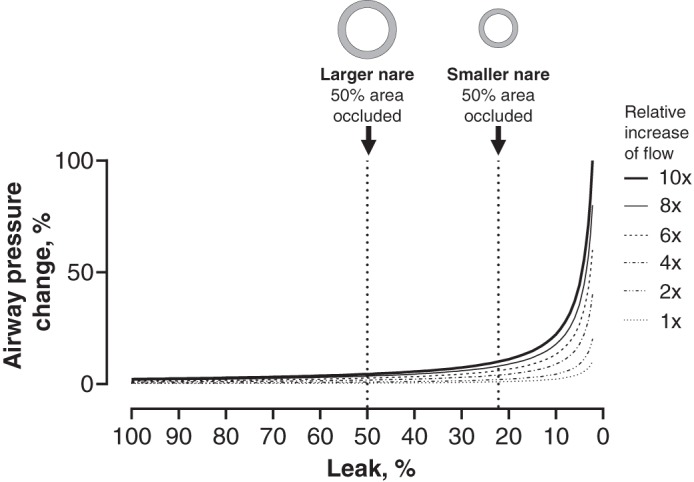
The graph shows the change in airway pressure, %, that may occur with a change in the leak area (%), as predicted by Poiseuille’s law, Q = π*r*^4^P/8η*L*, rearranged for pressure (P) as the solution and assuming that length (*L*) and fluid viscosity (η) remain the same across scenarios and are therefore removed so that the solution is dependent on the radius (*r*) and flow (Q). The law predicts that a high leak will result in low airway pressure regardless of the flow rate. As the area of the leak is reduced, the airway pressure will increase exponentially. For the same prong-to-nare ratio the airway pressure will be greater in the smaller nare because of a smaller leak area. Circles represent diameters of prongs and “nares,” and the gray area between the circles represents leak.

A model of the upper airway was utilized to understand how NHF generates positive airway pressure. The model replicates an ideal mouth-closed position, with the only leak being around the cannulas that were inserted into the “nares.” As the total occlusion increased, the flow rate had a greater effect on the airway pressure. During NHF, a reduced leak will increase the resistance to the gas flow exiting via the nares and airway pressure must increase, consistent with Poiseuille’s law. Figure 6 shows a conceptual relationship between airway pressure and the area of leak with Poiseuille’s law; if the leak is high, then the airway pressures are likely to remain low. As the leak is reduced, then the airway pressure may increase exponentially, which is shown in the results from the bench experiment. During NHF the flows are likely to be turbulent, and it should be noted that the law describes pressure changes in the laminar flow. Turbulent flow can help to explain the nonlinear effect of flow on PEEP observed in the bench results (Fig. 5).

The results demonstrate that the leak around the prongs is a primary determinant of airway pressures during NHF (7). NHF delivered via a smaller nare size versus a larger nare size will result in greater airway pressure for the same flow rate and prong-to-nare ratio because of the smaller leak area, as demonstrated in Fig. 6. Therefore, in smaller newborns with a low birth weight, a smaller cannula may still produce high pressures. The present findings address concerns raised by initial studies regarding the potential for a high level of positive airway pressure generated by delivering gas via nasal cannula (16). There is a very low possibility of very high positive airway pressure unless the nare is almost completely occluded. Changing the prong size can be used to adjust the airway pressure, which can potentially be titrated to patient requirements.

The mouth position may affect the pressures—an open mouth may increase the leak and decrease the pressure—as shown in the bench-top experiment (31). However, in the present study the lack of difference in pressure values between the open and closed positions of the mouth is inconsistent with other studies (14, 25). The results suggest that the position of the mouth, as observed, is not the only determinant of flow through the mouth and possibly the position of the soft palate may play an important role. The present bench model data need to be interpreted as in a mouth completely closed situation.

NHF augments the swings in airway pressure during breathing, probably by generating resistance to expiratory flow and reducing the resistance to inspiratory flow (23). In the present study bCPAP also increased the swings in airway pressure, which has been observed previously (18, 37). During bCPAP, the increased dynamic range of pressures may be caused by the high impedance in the breathing circuit. In adults, CPAP is produced by ventilators with low impedance, and with a low respiratory rate the fluctuation in airway pressures during breathing is smaller. Stable delivered pressure can be considered an advantage; however, CPAP is not typically used in adult patients with respiratory distress. The effect of the swings in airway pressure during NHF and bCPAP in treating respiratory distress in neonates deserves further research.

During bCPAP, the pressure in the nasal cavity was higher than the estimated pressure according to the user manual of 5.8 cmH_2_O for 8 L/min bias flow and 5 cmH_2_O set pressure in the water chamber. Kahn et al. demonstrated in premature infants that the measured pressure in the bCPAP interface is greater than the set pressure in the water chamber (13). Delivered pressure during bCPAP is always higher than the set pressure because of the resistance to flow in the interface and expiratory limb. This resistance causes a pressure differential between the interface and the distal end of the expiratory tubing that is proportional to the flow level applied (13).

### Limitations

The ventilation responses to NHF in adults are more variable when they are awake, and it is possible that a similar effect was observed during the first series of experiments (23). The pressure catheters inserted into each of the nares may have further compromised the breathing stability in the first part of the study. The second series of experiments included slightly more mature neonates, 39-wk GA (SD 1.5), who did not tolerate CPAP. Therefore, the authors were unable to further investigate the effects of CPAP on ventilation as was performed for NHF during the second part of the study.

It was not possible to measure PEEP accurately in the neonates, as this would require either breath holding or accurately measuring the breathing flows with a pneumotachometer to determine the end of expiration. Instead, the mean pressure and the dynamic range as a difference between peak expiratory and peak inspiratory pressure are reported. Data from the bench study, not shown, suggest that the mean airway pressure is very similar to the PEEP and may be used as a proxy measurement.

### Clinical Significance

The present findings indicate that minute ventilation in neonates is decreased in response to NHF whereas gas exchange is maintained. This is consistent with reduced rebreathing of expired gas, which is rich in CO_2_ and depleted in O_2_, from dead space (27). A reduction in the work of breathing by NHF, by reducing the required minute ventilation (2, 3, 27), may alleviate the symptoms of respiratory distress and reduce the need for the escalation of therapy. The findings help to explain recent evidence that NHF with room air only and no supplemental oxygen can successfully treat some infants with bronchiolitis (10). In response to NHF, the change in the respiratory rate should be interpreted with caution; the findings show that the required minute ventilation can still be significantly decreased even when the respiratory rate has increased.

The present results highlight the importance of the area of leak around the cannula and the flow rate as the fundamental mechanism by which NHF generates positive airway pressure. The data explain how pressure can be affected by mouth position (leak area) or body weight (nare size) and demonstrate that airway pressures may be adjusted by altering the prong size or flow rate. The relatively low nasal pressures at NHF of 8 L/min suggest a need to review the commonly used approach of NHF titration by 2 L/min per kilogram of body weight (11, 20) and may explain some unfavorable results of NHF therapy in neonates with low body weight who may require greater positive airway pressure (17, 24, 29).

### Conclusions

In newborns, NHF using heated and humidified room air substantially reduced minute ventilation without affecting gas exchange irrespective of a decrease or an increase of respiratory rate. NHF generates relatively low positive airway pressure that dynamically changes during the breathing cycle and exponentially increases with flow and occlusion of the nares. During the application of NHF in newborns with a standard cannula and flows of up to 8 L/min, pressure in the upper airways is unlikely to exceed 1–2 cmH_2_O. However, the pressure generated by NHF can be increased by reducing the leak around the cannula, which could be achieved by using larger prongs.

## GRANTS

Support for this study, by provision of NHF equipment and project costs, was provided by Fisher & Paykel Healthcare to P.M.

## DISCLOSURES

Materials for the study described in this article were supplied by Fisher & Paykel Healthcare. M.I.P. and S.T. are employees of Fisher & Paykel Healthcare. Other than the provision of equipment and financial support of project costs by Fisher & Paykel Healthcare, P.M. has no conflicts of interest, financial or otherwise, to disclose. M.D. has no conflicts of interest, financial or otherwise, to disclose.

## AUTHOR CONTRIBUTIONS

P.M., M.D., and S.T. conceived and designed research; P.M., M.D., M.I.P., and S.T. performed experiments; M.I.P. and S.T. analyzed data; P.M., M.D., M.I.P., and S.T. interpreted results of experiments; M.I.P. and S.T. prepared figures; M.I.P. and S.T. drafted manuscript; P.M., M.I.P., and S.T. edited and revised manuscript; P.M., M.D., M.I.P., and S.T. approved final version of manuscript.
